# The Maternal Health Care Crisis: Inequitable and Expensive∗

**DOI:** 10.1016/j.jacadv.2024.101069

**Published:** 2024-07-04

**Authors:** Melinda B. Davis, Oyinkansola Osobamiro

**Affiliations:** a Division of Cardiovascular Medicine, Department of Internal Medicine, University of Michigan, Michigan, USA; b Department of Obstetrics and Gynecology, University of Michigan, Michigan, USA

**Keywords:** cardio-obstetrics, cardiovascular, maternal care, maternal mortality, outcomes, pregnancy

**Figure:**
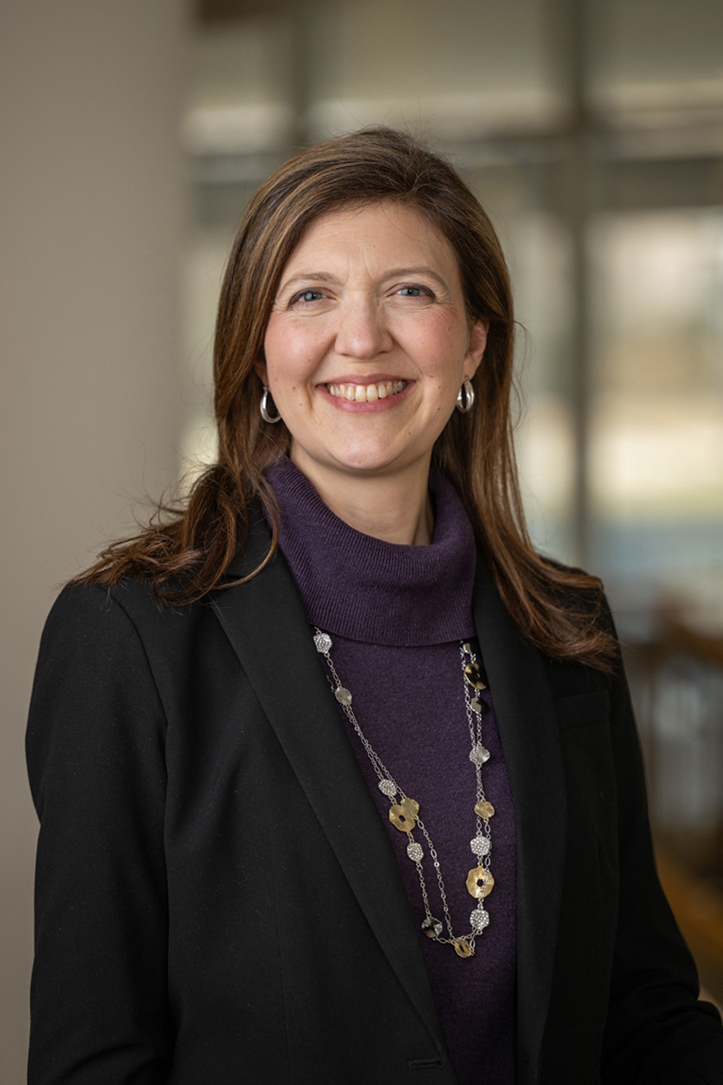

Cardiovascular disease (CVD) is the leading cause of maternal morbidity and mortality in the United States. Compared to similarly wealthy nations, the United States has the highest maternal mortality, and this continues to rise.^1^ Moreover, the burden of poor maternal outcomes is disproportionally shared by the most vulnerable. Pregnant women who experience racism, have lower incomes, have limited insurance options/public insurance, live in rural areas, or have poor social support are significantly more likely to have CVD during pregnancy and major adverse cardiovascular events (MACE).^2^ As these socioeconomic risk factors accumulate, outcomes worsen.

A well-documented irony of the American health care system is that health care costs remain incredibly high, despite poor outcomes. Maternal morbidity and mortality are no exception. Health care for a single normal pregnancy is estimated to cost nearly $19,000 overall.^3^ With any pregnancy complication, these costs rise substantially due to extended hospitalizations and additional interventions.^4^ Nonmedical costs, such as lost work productivity, further contribute to the societal costs of maternal morbidity and mortality.

In this issue of *JACC: Advances*, Williamson et al^5^ tackle risk factors for adverse cardiovascular outcomes in pregnancy, as well as the increased financial costs of caring for this vulnerable population. This study is one of the larger retrospective studies examining risk factors for adverse cardiovascular outcomes. The authors conducted a retrospective cohort study of pregnant people ages 18 to 50 using data from the National Inpatient Sample Database. Patients included in the CVD cohort had a diagnosis of congenital heart disease, valvular heart disease, cardiomyopathy, or arrhythmias by international classification of disease codes. Notably, aortopathies were not included in this cohort. Pregnant patients with CVD were overall older, predominately White (with the exception of the subset with cardiomyopathy), and had increased comorbidities, multifetal pregnancies, and cesarean births. MACE were defined as cardiac death, cardiac arrest, heart failure, myocardial infarction, or vascular injury. Malignant arrhythmias were not included as an outcome.

Patients with CVD experienced worse cardiovascular outcomes and had higher obstetric and neonatal complications. Patients with cardiomyopathy and ischemic heart disease had 50-fold and 30-fold greater odds, respectively, of MACE than those without CVD. This study also noted that Black race, low income, and use of public insurance were independently associated with higher rates of MACE, despite the fact that the majority of patients within the study cohort were White. This association further highlights that socioeconomic risk factors alone increase a patient’s risk of significant adverse cardiovascular outcomes during pregnancy. The association of MACE with public insurance like Medicaid is particularly interesting, though possible explanations for this are not fully explored in the study. Given that one must have an income at or below 138% of the federal poverty level to qualify for Medicaid, prior poor access to care may lead to adverse sequelae during pregnancy, such as suboptimally controlled diabetes and hypertension. In the Canadian-derived CARPREG II (Cardiac Disease in Pregnancy II) risk index, late prenatal care conferred additional risk for cardiovascular complications.^6^ Although the study by Williamson et al only examined in-hospital complications, significant maternal morbidity and mortality occur in the postpartum period; therefore, continuation of comprehensive health insurance is essential for decreasing complications and future CVD risk.^7^

A novel aspect of this study is the analysis of health care costs associated with cardiovascular and obstetric complications at the time of delivery. This is one of the few studies to specifically focus on CVD at the time of delivery. Complications among patients with CVD resulted in a staggering total of $1,075,000,000 in health care expenditures, with $237 million spent on MACE alone. Patients with cardiomyopathy had the worst outcomes, with a length of stay of 5.37 days and an average hospitalization cost of $15,341, nearly three times the cost for patients without CVD. Those with ischemic heart disease followed close behind, with an average hospitalization cost of $11,404. To put this in context, these costs represent a single snapshot at the time of the delivery hospitalization.

The authors should be commended for highlighting the significant economic cost of the delivery hospitalization for patients with CVD. Additional research is needed to determine why these costs are so high, how they can be reduced, and, more importantly, how patient outcomes could be improved. Some areas to explore include whether costs and outcomes vary depending on the size or type of hospital, the presence or absence of a multidisciplinary cardio-obstetrics program, urban vs rural settings, and the type of prenatal counseling and antepartum surveillance. Significant costs come from cesarean deliveries, monitoring in intensive care units, and increased lengths of stay. Many practice patterns are based on local institutional experience and expert opinion; however, increased costs may not translate to improved clinical outcomes. For instance, planned cesarean deliveries are likely overutilized since the majority of patients with CVD can labor safely, even those with cardiomyopathies.^8^ As the field of cardio-obstetrics continues to evolve, standardization of best practices and the creation of Cardio-Obstetrics Centers of Excellence could reduce unnecessary health care expenditures.

Examining MACE through a health economics lens lends further urgency to the maternal cardiovascular health crisis and offers opportunities for health policy intervention. While Williamson et al focused primarily on health care costs during delivery, this may not reflect the period of highest health care expenditure. Of pregnancy-related deaths, 31% occur during pregnancy, 17% occur on the day of delivery, and the remaining majority occur in the postpartum period.^9^ Postpartum costs would include rehospitalizations, procedures, and lost wages from extended time away from work. In addition to postpartum costs, we lack data about the costs of managing patients with CVD throughout pregnancy, such as preconception counseling, prenatal care, testing, and management. Frequent clinical surveillance and active management of patients in a cardio-obstetrics program could potentially reduce the costs of care and MACE at the time of delivery. For example, if patients with cardiomyopathy—who in this cohort were more likely to be Black, a demographic risk factor independently associated with worse cardiovascular outcomes—received more frequent monitoring with visits and echocardiograms throughout their pregnancy, would this decrease the incidence of MACE? If so, would the decrease in MACE-related health care and nonhealth care expenditures offset the increased costs of more frequent monitoring? Further research is needed to better understand the most effective method for monitoring high-risk birthing people during preconception, throughout pregnancy, and in the postpartum period to improve both clinical outcomes and reduce reactionary health care spending.

Pregnancy is a unique opportunity to engage women within the health care system. Given that reproductive rights are no longer nationally guaranteed, more birthing people with high-risk CVD will likely interact with the health care system, and an increased percentage will have one or more socioeconomic risk factors for adverse outcomes. The current study underscores the clinical and economic urgency to determine optimal care and delivery plans for cardio-obstetrics patients. As illustrated in this study, we must continue to strive for more effective methods of reducing adverse maternal outcomes, while also providing more equitable affordable care.

## Funding support and author disclosures

The authors have reported that they have no relationships relevant to the contents of this paper to disclose.
